# The differential time course for consonant and vowel processing in Arabic: implications for language learning and rehabilitation

**DOI:** 10.3389/fpsyg.2014.01557

**Published:** 2015-01-22

**Authors:** Sami Boudelaa

**Affiliations:** ^1^Department of Linguistics, Faculty of Humanity and Social Sciences, United Arab Emirates UniversityAl Ain, United Arab Emirates; ^2^Department of Psychology, University of CambridgeCambridge, UK

**Keywords:** consonantal root, vocalic word pattern, time course of spoken word processing, CV-hypothesis, learning and rehabilitation

## Abstract

Educators and therapists in the Arab world have not been able to benefit from the recent integration of basic behavioral science with neuroscience. This is due to the paucity of basic research on Arabic. The present study is a step toward establishing the necessary structure for the emergence of neuro-rehabilitory and educational practices. It focuses on the recent claim that consonants and vowels have distinct representations, carry different kinds of information, and engage different processing mechanisms. This proposal has received support from various research fields, however it suprisingly stops short of making any claims about the time course of consonant and vowel processing in speech. This study specifically asks if consonants and vowels are processed differentially over time, and whether these time courses vary depending on the kind of information they are associated with. It does so in the context of a Semitic language, Arabic, where consonants typically convey semantic meaning in the form of *tri-consonantal roots*, and vowels carry phonological and morpho-syntactic information in the form of *word patterns*. Two cross-modal priming experiments evaluated priming by fragments of consonants that belong to the root, and fragments of vowels belonging to the word pattern. Consonant fragments were effective primes while vowel fragments were not. This demonstrates the existence of a differential processing time course for consonants and vowels in the auditory domain, reflecting in part the different linguistic functions they are associated with, and argues for the importance of assigning distinct representational and processing properties to these elements. At broader theoretical and practical levels, the present results provide a significant building block for the emergence of neuro-rehabilitory and neuro-educational traditions for Arabic.

## Introduction

Neuro-rehabilitation and neuro-education are two nascent scientific disciplines that are informed by research from cognitive neuroscience and behavioral psychology (Taub et al., 2002; Devonshire and Dommett, 2010; Ansari et al., 2012; Hook and Farah, 2012). The main aim of neuro-rehabilitation is to ameliorate dysfunctional cognitive and brain functions caused by disease or injury (Robertson and Fitzpatrick, 2008; Nehra et al., 2014). In contrast, neuro-education seeks to create a better understanding of how we learn and how knowledge about the functional properties of the brain can be harnessed to create more effective teaching methods, curricula, and educational policies (Hardiman et al., 2009; Carew and Magsamen, 2010). Despite their relatively recent history both disciplines are making significant strides toward helping with rehabilitory and educational processes. This success has been made possible thanks to the burgeoning fields of cognitive neuroscience and behavioral psychology. For example, recent research has revealed the existence of “neural markers” of learning disorders, most notably in the case of dyslexia. Imaging studies have revealed that human infants at risk of dyslexia (i.e., with immediate family members who suffer from dyslexia) show atypical neural responses to changes in speech sounds, even before they are able to understand the semantic content of language (Leppänen et al., 2002). Such a finding allows for the early identification and remediation of potential learning disorders.

Unfortunately however the blossoming of cognitive neuroscience and behavioral psychology holds true only of certain regions in the world such as Europe and North-America. Other geographic areas, in particular the Arab world, suffer from a pronounced dearth of basic research which has led to serious deficiencies in effective interventions in neurorehabilitation and a complete lack of a neuro-educational culture.

The purpose of the present paper is to make an initial contribution to developing the necessary building blocks from basic psycholinguistic research for the emergence of neuro-rehabilitory and neuro-educational practices in the Arab world. More specifically, the paper reports two psycholinguistic experiments aiming at determining how information about consonants and vowels is derived from the auditory input and mapped onto lexical knowledge. The results, as we will argue in the general discussion, can inform practitioners both in rehabilitation and education.

## Consonants and vowels in language processing

A long-standing debate in cognitive science relates to what levels of representations are available to and used by the language processing system. In the context of this general debate, many studies have come to focus on the status of consonants and vowels, asking whether these are categorically distinct objects that are independently represented and differently processed (Caramazza et al., 2000; Nespor et al., 2003, Bonatti et al., 2005, 2007; Nazzi, 2005; Mehler et al., 2006; Knobel and Caramazza, 2007; Toro et al., 2008), or whether they are simply convenient labels that distinguish sonority peaks (vowels) from sonority troughs (consonants) in the speech stream and need neither to be interpreted as distinct constructs, nor to invoke different processing mechanisms (Monaghan and Shillcock, 2003, 2007; Keidel et al., 2007).

The existing data relating to this debate derive exclusively from research into Indo-European languages. Caramazza et al. (2000) report data from two Italian patients who showed contrasting selective difficulties in producing vowels and consonants. These results were taken as evidence that consonants and vowels are independently represented. However, this interpretation was challenged by Monaghan and Shillcock (2003, 2007), who argued that the double dissociation between vowels and consonants can be modeled as an emergent effect of modular processors operating on feature-based representations with no need to posit separate representations for vowels and consonants.

Bonatti et al., (2005) report a study in which French speaking subjects learned an artificial language where words were strings of alternating consonants (C) and vowels (V) (e.g., ^*^puragi), and were asked to indicate in a forced choice test which items belonged to the artificial language. Subjects picked up on the regularities for consonants, but not vowels suggesting that the two elements engaged different processing mechanisms. Keidel et al. (2007) challenged this interpretation and contended that differences in the distribution of consonants and vowels in French may explain why French speakers pick up on the regularities provided by consonants but not those provided by vowels.

Keidel et al.'s criticism was addressed in a subsequent study by Toro et al. (2008), who extended Bonatti et al.'s, findings to Italian speakers. Specifically, having learnt a nonsense set like ^*^badeka, ^*^bedake, where the vowel structure is ABA, Italian speakers preferred sequences like ^*^biduki, ^*^budiku, with the same ABA vowel structure, although the vowel sequences *-i-u-i or -u-i-u* were never part of the familiarization stream. Importantly, the same subjects were unable to extract comparable generalizations using consonants from sequences like ^*^benobu and ^*^pikeko.

The distinction between consonants and vowels at the cognitive level has some support at the neural level. For instance, neuropsychological research offers descriptions of lesions in left temporal, parietal and fronto-parietal regions or bilateral parietal cortex which affect consonants and vowels differentially (Caramazza et al., 2000). Similarly, electrophysiological evidence broadly suggests anterior-posterior dissociation for consonants and vowels, respectively. For instance, Carreiras et al. (2007) showed that correct NO responses to pseudowords during lexical decision evoked N400 effects in anterior (F5 line) and middle regions (C5 line) when consonants are transposed, but in middle and posterior regions (P5 line) when vowels are transposed. However, there was no clear lateralization associated with these effects. A more fine-grained localisation is provided using PET (Sharp et al., 2005). In Sharp et al.'s study participants were asked to generate real words from heard pseudowords created by the substitution of either a vowel or consonant. When consonants needed to be substituted, word generation was more difficult and left inferior frontal activation was higher. However, there was no increase of activation for vowels relative to consonants in the left suggesting that that vowel processing may share neural resources with prosodic processing in the right hemisphere. More recently Carreiras and Price (2008) used functional magnetic resonance imaging to investigate whether vowel and consonant processing differences are expressed in the neuronal activation pattern and whether they are modulated by task. The tasks used were reading aloud and lexical decision on visually presented pseudowords created by transposing or substituting consonants and vowels in real words. In the reading aloud task, changing vowels relative to consonants increased activation in a right middle temporal area typically associated with prosodic processing of speech input. In contrast, in the lexical decision task, changing consonants relative to vowels increased activation in right middle frontal areas typically associated with response inhibition. The task-sensitive nature of these effects underscores the fact that consonants and vowels place differential processing demands on the brain and differentially engage various neural structures.

These results provided the basis for the development of the Consonant Vowel-hypothesis (CV-hypothesis) which holds that consonants and vowels fulfill different roles across languages with consonants carrying lexical information and vowels encoding morpho-syntactic and phonological information. These two elements also engage two processing mechanisms. The first relies on transitional probabilities between consonants to extract words and access the lexicon for meaning, while the second relies on the structure defined by vowels to draw generalizations about the input (Nespor and Vogel, 1986; Nespor et al., 2003, Bonatti et al., 2005; Nazzi, 2005; Toro et al., 2008).

The CV-hypothesis has far-reaching implications. Not least, it implies that the language system consists of different levels of representations each with its own internal structure, and each with its specialized computational requirements. Although these implications impose stringent constraints on the dynamics of online spoken word recognition, the CV-hypothesis remains highly underspecified in this respect. Specifically, it says nothing about the potential timing differences underlying the uptake of information about consonants and vowels from speech. For the proper development of the CV-hypothesis as a speech processing model, it is essential to specify the dynamics of the speech mapping process.

The present paper aims to fill this gap (a) by developing, within the CV-hypothesis framework, a set of specific claims about the time course underlying the projection of consonantal and vocalic information onto lexical representations, and (b) by empirically testing these claims. To do this, one needs to evaluate consonants and vowels in languages where the distinction between these two elements is not limited to the phonological domain, but has overt implications for meaning. Semitic languages like Arabic and Hebrew offer this opportunity, with the consonant-vowel contrast being overtly relevant at morphological, semantic and syntactic levels (McCarthy, 1981). Consequently this study focuses on Arabic root consonants as bearers of semantic meaning, and vocalic patterns as carriers of phonological and morpho-syntactic information. In so doing it will take our understanding of the cognitive architecture subserving consonant-vowel processing to a new level of generality by exploring the Semitic system, while achieving at the same time new levels of specificity by uncovering potentially different processing procedures across languages.

## Consonants and vowels in a semitic context

The consonant-vowel contrast in Semitic languages defines two functionally distinct morphemes, with consonants making up the *root*, and vowels corresponding to the *word pattern*^1^. The root is typically comprised of 3 consonants and conveys the general meaning which will be present to various degrees in all other words featuring that root. By contrast the word pattern is a composite morpheme with derivational and inflectional functions (Prunet et al., 2000; Boudelaa and Marslen-Wilson, 2004, 2005). More specifically, the word pattern is like a template that determines not only the overall shape of the surface form, its phonological structure and its stress pattern, but it is also the bearer of morpho-syntactic information such as *active*, *passive*, *plural*, etc. Both the root and the word pattern are bound morphemes and cannot surface unless they are interleaved within each other nonlinearly. For example the Arabic root {ktm} with the general meaning of *hiding*, and the pattern {-a-a-} with a *past tense active* meaning are interleaved to generate the surface form [katam] *hide, active, perfective, verb*.

In such a linguistic environment, it makes sense that consonants and vowels are segregated since downstream processing relies on such distinct parts of the word. This raises the question of whether this segregation results in a differential uptake of information about roots (consonants) and word patterns (vowels) over time.

## The CV-hypothesis in a semitic context

Spoken word recognition is a rapid process, typically taking less than a quarter of a second to complete (Pulvermüller et al., 2006; Hauk et al., 2009). For this reason, it is generally thought that the process of mapping speech input onto internal representations is governed by the principle of maximal processing efficiency, whereby the incoming speech is analyzed at all points and the most informative output available is derived from it (Marslen-Wilson and Welsh, 1978; Marslen-Wilson and Tyler, 1980; Pirog Revill et al., 2008a,b). If, as the CV-hypothesis claims, the recognition process is oriented toward consonants which are used to extract information about word identity, consonants should be processed continuously such that at each point in time where the speech input contains a consonant, a lexical access process is initiated and the best fitting candidates are activated. In the context of priming, this means that a fragment of an Arabic root should be an effective prime of a target sharing the same root consonants. This prediction is tested in Experiment 1.

Another important feature of the CV-hypothesis is that vowels are used to derive generalization about the linguistic environment. Generalization is the notion that humans are able to respond in a similar way to different stimuli provided those stimuli have similar properties. In this respect, evoking the same response to the Arabic words [katab] *write*, and [daxal] *enter*, based on their similar vowel patterns is an instance of generalization. Arguably, in order to derive this kind of generalization one needs to hear the full sequence. If correct, this predicts that information about vowels should not be mapped continuously because the language processor needs to accumulate enough information before it can draw reliable generalizations about the overall structure of the word (Lahiri and Marslen-Wilson, 1992). In a priming context, this translates into the prediction that a fragment of an Arabic word pattern should be less effective as a prime than the full word pattern. Experiment 2 tests this prediction.

## Experiment 1

Is information about root consonants mapped continuously onto internal representations of lexical form as the CV-hypothesis predicts? This question is tackled using the cross-modal priming paradigm in which participants have to make a speeded lexical decision about a visual target presented immediately at the offset of an auditory word-fragment prime or a full word prime. Since the prime is auditory and the target is visual in this paradigm, any savings in the processing of the target should be attributed to repeated access of the same underlying modality-independent representation. If priming obtains between words sharing a root fragment, this will suggest that the consonantal root is represented as an independent unit, and that information about it is continuously evaluated, segment by segment as that information becomes available.

### Method

#### Participants

Eighty one volunteers (50 females) aged 16 to 20 were tested. They were students at the high school of Tataouine in the South of Tunisia. The subjects were native MSA speakers and studied French and English as second and third languages. None of them had any history of hearing loss or speech disorders. Written consent to take part in the study was obtained either from the participants themselves or from their guardians if they were minors. The study was approved by the Peterborough and Fenland Ethical Committee.

#### Materials and design

Forty-eight orthographically unambiguous targets were used. They were on average 4.58 letters long (*SD*: 0.66), 8 phonemes long (*SD*: 1.25), and 3.33 syllables long (*SD*: 0.48). Ten different word patterns were used to construct this set of words, which was divided into two subsets of 24 words each, matched on length and frequency checked using the ARALEX database (Boudelaa and Marslen-Wilson, 2010). Each target in the first set was paired with four types of primes as outlined in Table 1.

**Table 1:** **Examples of stimuli used in the different conditions of experiment 1**.

	**Prime**	**Target**
	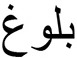	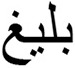
1a: Root, Full Prime	[buluu 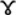 un] *puberty*	[balii 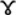 un] *eloquent*
	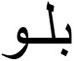	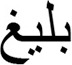
1b: Root, Partial Prime	[buluu 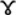 un]	[balii 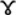 un] *Eloquent*
	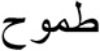	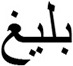
1c: Baseline, Full Prime	[t^ 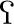 ^umuuħun] *Ambition*	[balii 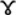 un] *Eloquent*
	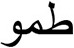	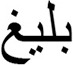
1d: Baseline, Partial Prime	[t^ 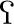 ^umuu]	[balii 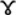 un] *eloquent*
	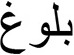	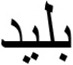
2a: Phon, Full Prime	[buluu 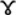 un] *puberty/reaching*	[baliidun] *silly*
	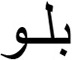	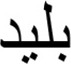
2b: Phon, Partial Prime	[buluu]	[baliidun] *silly*
	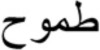	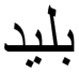
2c: Baseline, Full Prime	[t^ 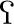 ^umuuħun] *Ambition*	[baliidun] *silly*
	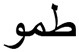	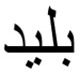
2c: Baseline, Partial Prime	[t^ 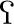 ^umuu]	[baliidun] *silly*

Examples are given in Arabic script with a phonetic transcription and an English gloss.

In the +Root, Full Prime condition, the priming word (e.g., [buluu
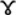
un] *puberty*), and the target (e.g., [balii
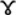
un] *eloquent*) share the consonants of the root {bl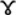} and the prime is presented in full. In condition 1b, labeled +Root, Partial Prime, the same target [balii
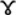
un] is paired with the fragment [buluu] excised from the full prime [buluu
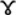
un]. Note that the only shared material across prime and target are consonants. To provide appropriate controls against which to measure priming, the target [balii
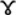
un] was paired with an unrelated full prime [t^
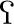
^umuuħun] *ambition* in the Baseline Full Prime condition and with the unrelated fragment [t^
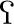
^umuu] taken from the full prime [t^
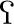
^umuuħun] in the Baseline Partial Prime condition.

To ensure that the partial primes are ambiguous and can be the beginning of different words in the language, the first three to four segments of each full prime in conditions 1a and 2a were excised and presented in a simplified gating task to 15 subjects from the same linguistic background and same age range as those who took part in the priming experiment (Grosjean, 1980). In this task subjects are typically presented with successive auditory fragments of 50 ms long, and they are instructed to suggest a word that can be a continuation to the fragment being presented, and to say how confident they are of their guess. Each partial prime was on average 175 ms long (SD: 25.31), and was presented incrementally in steps of 50 ms. At each step subjects had to guess the word and to say how confident they were of their guess on a scale from 10 *completely sure* to 1 *completely unsure*. For each fragment, subjects suggested on average 11.41 possible different words, (8.12 different roots), and their confidence ratings (their degree of certainty about the word they suggested) were low, averaging 4 on a 10-point scale. Taken together the large number of suggested words and the low confidence ratings suggest that the fragment of the word the subjects were exposed to was ambiguous enough to match or activate different possible lexical candidates.

The 24 targets in the second set were also paired with the same set of primes to form a phonological control condition. In other words, the primes were constant but the targets were different. Phonological overlap is defined in this study as the number of shared phonemes from onset between a given pair of words. This is illustrated in the +Phon, Full Prime and the +Phon, Partial Prime conditions where the word [buluu
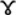
un] *puberty*, and its fragment [buluu] are used to prime the phonologically related target [baliidun] *silly*. This target is phonologically related to the partial prime [buluu] and provides a viable continuation to its consonantal structure. However, it features the root {bld}, which is different from the root {bl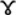} underlying the full-prime [buluu
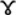
un] *puberty*. The control prime [t
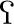
umuuħun], used in the Baseline, Full Prime condition, and its fragment [t
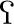
umuu] used in the Baseline, Partial Prime condition, provide baseline conditions for evaluating priming in the +Phon, Full Prime and the +Phon, Partial Prime.

Primes and targets shared on average 57.3% of their phonemes in condition 1a condition, 44.6% in condition 1b, 56.3% in condition 2a, and 47.9% in condition 2b. Seventy-two unrelated word-word pairs were included to reduce the proportion of related pairs in the experiment to 20%. Half of these had a partial prime and half a full prime. Another 120 word-nonword pairs with similar characteristics as the word-word pairs were used to to provide the nonword targets needed for the lexical decision task employed here. Forty practice trials that were representative of the experimental trials were used. To avoid repetition of primes and targets within subjects, four counterbalanced experimental lists were constructed each consisting of 280 pairs.

#### Procedure

The prime words were recorded by a native speaker of Arabic and digitized with a sampling rate of 44 kHz. Subjects heard the stimuli at a comfortable level through HD 250 Sennheiser headphones. The sequence of stimulus events within each trial started with a 1000 ms silence followed by an auditory prime. Immediately at the offset of the prime a visual target was displayed on the screen for 2000 ms. Timing and response collection were controlled by a laptop PC running the DMDX package (Forster and Forster, 2003). Participants were instructed to make a lexical decision as quickly and as accurately as possible. The experiment, which lasted for 35 min, started with the practice trials followed by the rest of the stimuli.

## Results and discussion

Error trials were excluded and not replaced (2.66%). Outlying responses above 3000 ms or below 100 ms were also excluded (0.07%). The remaining data were inverse transformed (multiplied by 1/1000) to reduce the influence of outliers (Ratcliff, 1993). Table 2 gives the percent error rates and the means of the reaction times.

**Table 2:** **Reaction times, (standard deviations), amount of priming, and %error rates for the target preceded by test and baseline primes in the different conditions of experiment 1**.

**Condition**	**Test**	**Baseline**	**Priming**	**Error in Test**	**Error in Baseline**
+Root, Full Prime	557 (41)	595 (52)	38	1.74	2.23
+Root, Partial Prime	568 (47)	602 (50)	34	3.10	2.71
+Phon, Full Prime	609 (55)	604 (64)	−5	3.10	2.72
+Phon, Partial Prime	583 (53)	600 (63)	17	3.29	2.33

Two mixed design analyses of variance (ANOVAs) across subjects (F1) and across items (F2) were conducted on the reaction time and accuracy data. They included the factors Condition (morphology vs. phonology), Prime Type (related vs. unrelated), and Prime Length (full prime vs. partial prime). Condition was treated as a repeated factor in the participants' analysis and as an unrepeated factor in the items analysis, while Prime Type was treated as a repeated factor in both analyses. A fourth variable “List” was also included in the analyses as a dummy variable to reduce the estimate of random variation (Pollatsek and Well, 1995). This variable was treated as a between-subjects factor in the participants analysis and as a between-items factor in the items analysis. The *p*-values reported for the ANOVA in this an the next experiment are adjusted with the Greenhouse–Geisser epsilon correction for nonsphericity. There were significant main effects of the factors Condition [*F*_1(1, 80)_ = 37.43, *p* < 0.0001; *F*_2(1, 47)_ = 10.52, *p* < 0.0022] and Prime Type [*F*_1(1, 80)_ = 59.16, *p* < 0.001; *F*_2(1, 47)_ = 14.76, *p* < 0.001]. The main effect of Prime Length was not significant [*F*_1_ and *F*_2_ < 1]. Condition interacted significantly with Prime Type [*F*_1(1, 80)_ = 25.14, *p* < 0.001; *F*_2(1, 47)_ = 17.71, *p* < 0.05], and Prime Length [*F*_1(1, 80)_ = 18.38, *p* < 0.001; *F*_2(1, 47)_ = 15.76, *p* < 0.005]. The two-way interaction between Prime Type and Prime Length was not significant [*F*_1_ < 1; *F*_2_ < 1]. The theoretically important three-way interaction between Condition, Prime Type and Prime Length was significant [*F*_1(2, 80)_ = 6.18, *p* < 0.01; *F*_2(2, 47)_ = 10.52, *p* < 0.01], indicating that priming was not constant across the different conditions. Further planned comparisons using 0.05 Bonferroni protection levels confirmed this (Keppel, 1982). Priming was significant in the [+Root, Full Prime] case [*F*_1(1, 80)_ = 49.36, *p* < 0.001; *F*_2(1, 23)_ = 10.94, *p* < 0.001], the [+Root, Partial Prime] case [*F*_1(1, 80)_ = 39.83, *p* < 0.001; *F*_2(1, 23)_ = 8.61.52, *p* < 0.001], and the [+Phon, Partial Prime] case [*F*_1(1, 80)_ = 7.27, *p* < 0.05; *F*_2(1, 23)_ = 6.65, *p* < 0.05], but not in the [+Phon, Full Prime] case [*F*_1_ < 0.05; *F*_2_ < 1]. Furthermore, the magnitude of priming in the [+Root, Full Prime] was not significantly different either from that in the [+Root, Partial Prime] case or the [+Phon, Partial Prime] case [all *F*s< 1]. By contrast there was a reliable difference between the amount of priming observed in the [+Phon, Full Prime] condition and (a) the [+Root, Full Prime] condition [*F*_1(1, 80)_ = 6.86, *p* < 0.05; *F*_2(1, 23)_ = 5.04, *p* < 0.05], (b) the [+Root, Partial Prime] condition [*F*_1(1, 80)_ = 5.91, *p* < 0.05; *F*_2(1, 23)_ = 0.059], and (c) the [+Phon, Partial Prime] condition [*F*_1(1, 80)_ = 7.12, *p* < 0.05; *F*_2(1, 23)_ = 6.18, *p* < 0.05]. Similar statistical analyses were conducted on the error data but no across conditions differences were found.

Finally, to check on the possible contribution of two stimulus properties—the acoustic duration of the prime, and the degree of phonological overlap between prime and target—each of these variables was centered and used as a predictor of priming in separate stepwise multiple regression analyses. Neither duration [*R*^2^ = 0.003, *F*_(1, 94)_ = 0.26, *p* = 0.60] nor phonemic overlap [*R*^2^ = 0.004, *F*_(1, 94)_ = 0.34, *p* = 0.55] was a significant predictor of priming.

This experiment suggests that full word primes and targets sharing a consonantal root prime each other reliably, while phonologically related full primes fail to do so. This is consistent with a continuous view of spoken word recognition, but it does not rule out the possibility of discontinuous processing whereby the language processor waits until the whole three consonants of the root are heard before it attempts lexical access. This interpretation is ruled out however by the effects for partial primes. In particular, partial primes such as [buluu] are as effective as the complete word [buluu
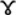
un] in priming lexical decision to a probe with which they share the same consonants (e.g., [balii
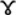
un] *eloquent*; [baliidun] *silly*). This suggests that the lexical processor attempts to find a lexical match as soon as sufficient consonantal information is extracted from the speech stream. Upon hearing the partial prime [buluu] many word candiates whose roots contain the consonants {b,l} (e.g., {bl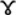}, {bll}, {bl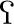}, {bly}) are activated and start competing for recognition. Otherwise there would be no basis for the comparable facilitation observed in the +Root Partial Prime condition and the +Phon Partial Prime condition. In summary, the results of Experiment 1 suggest that information about the consonantal root is continuously evaluated as the relevant information becomes available in the speech stream.

## Experiment 2

This experiment asks whether word patterns, like roots, are continuously mapped onto the lexicon. It co-varies two factors: (a) the prime and target relationship such that they share either a word pattern or a phonological overlap, and (b) prime length using full words or fragments of words as primes. To derive predictions for this experiment, two claims needs to be allied. The first is the CV-hypothesis claim that vowels (word patterns) are used to draw generalizations about the structure of the input. The second is the claim that generalizations can be successfully drawn only if the appropriate information is available. Thus, the question is whether partial information about the Arabic word pattern is enough to identify the correct pattern and trigger lexical access.

### Method

#### Participants

Eighty-eight volunteers (40 famles) from the same age range and background as those in Experiment 1 were tested.

#### Materials and design

Forty eight orthographically unambiguous words were chosen to serve as targets. They consisted on average of 3.54 letters (*SD*: 0.85), 3.38 syllables (*SD*: 0.73) and 6.06 phonemes (*SD*: 1.59). Fourteen different word patterns were used to construct these items, which were divided into two sets of 24 words matched on length, and frequency, checked using the ARALEX database. Each target in the first set was paired with four types of primes as illustrated in Table 3.

**Table 3:** **Sample stimuli used in the different conditions of experiment 2**.

	**Prime**	**Target**
	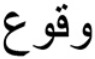	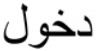
1a: +WP, Full Prime	[wuquu 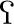 un] *happening*	[duxuulun] *entering*
	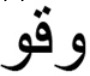	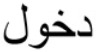
1b: +WP, Partial Prime	[wuquu]	[duxuulun] *entering*
	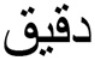	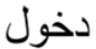
1c: Baseline, Full Prime	[sufunun] *accurate*	[duxuulun] *entering*
	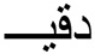	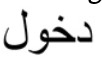
1d: Baseline, Partial Prime	[daqii]	[duxuulun] *entering*
	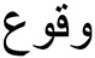	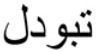
2a: +Phon, Full Prime	[wuquu 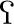 un] *happening*	[tubuudila] *to be exchanged*
	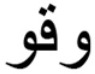	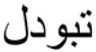
2b: +Phon, Partial Prime	[wuquu]	[tubuudila] *to be exchanged*
	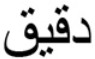	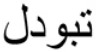
2c: Baseline, Full Prime	[daqiiqun] *accurate*	[tubuudila] *to be exchanged*
	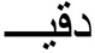	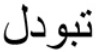
2c: Baseline, Partial Prime	[daqii]	[tubuudila] *to be exchanged*

Examples are given in Arabic script with a phonetic transcription and an English gloss.

In the +WP, Full Prime condition, the prime is a full word (e.g., [wuquu
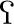
un] *happening*) that shares the vowels of the word pattern (e.g., {u-uu-} *perfective, active*) with the target (e.g., [duxuulun] *entering*). In the +WP, Partial Prime condition, the same target is paired with a fragment (e.g., [wuquu] excised from the full prime [wuquu
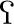
un]. The fragment primes were on average 238 ms long (*SD*: 38). In a gating task run on these fragments (see Experiment 1 for details), 15 subjects who did not participate in the priming experiment suggested on average 11.97 possible different words (with 3.6 different word patterns on average). Their confidence ratings were generally low averaging 3.5 which means that the fragmentary primes were compatible with many lexical hypotheses. The Baseline, Full Prime and the Baseline, Partial Prime conditions use a full word (e.g., [daqiiqun] *accurate*) and a fragment of it (e.g., [daqii]) as respective baseline primes for the +WP, Full Prime and the +WP, Partial Prime conditions.

To provide a phonological control condition which assesses form overlap from sequence onset, the second set of 24 targets was paired with the same set of prime words as illustrated in Table 3. The +Phon, Full Prime condition with prime-target pairs like [wuquu
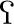
un]-[tubuudilaa] *happening-to be exchanged*, assesses the extent to which pure phonological overlap in the sense of sharing a number of vowel segments that do not make up the same morpheme can be facilitatory. The amount of vocalic overlap in this condition is the same as that in the +WP, Full Prime condition. However, in the +WP, Full Prime condition, the shared vowels make up the same nominal morpheme with singular meaning in the prime and target, while in the +Phon, Full Prime condition, the vowels in the target are in the context of a verb and convey a passive perfective meaning. In the +Phon, Partial Prime condition, the fragment [wuquu] excised from the full prime [wuquu
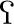
un] *happening* is paired with the target [tubuudila]. The question here is whether partial phonological overlap can trigger access to the related target. Finally the target [tubuudila] is paired with the unrelated full prime [daqiiqun] *accurate* in the Baseline, Full Prime condition, and by its fragment [daqii] in the Baseline, Partial Prime condition. In this experiment, prime and target pairs shared on average 62.87% of their phonemes in Condition 1a, 34.53% in condition 1b, 49.80% in condition 2a, and 43.32% in condition 2b.

The numbers of word-word and word-nonword fillers used were similar to those in Experiment 1. The prime-target relatedness proportion was kept at 20%. Additionally, 40 practice trials that were representative of the experimental trials were selected. Four counterbalanced experimental lists were constructed each consisting of 280 pairs.

#### Procedure

This was identical to the procedure for Experiment 1.

## Results and discussion

The data for 2 participants were rejected because of high error rates (above 12%). Error trials were excluded (3.51%). Cut-offs were set at 3000 ms and below 100 ms and excluded only 0.05% of the data. The remaining data were inverse transformed to reduce the effects of outliers. Item and participant means were then calculated (see Table 4) and analyzed as before using the variables Conditions (morphology vs. phonology), Prime Type (related vs. unrelated), Prime Length (full prime vs. partial prime). A dummy variable representing either the participants grouping in the allocation of subjects to experimental list or the test item grouping in the allocation of items to lists, was included to reduce the estimate of random variation.

**Table 4:** **Reaction times, (standard deviations), amount of priming, and %error rates for the target preceded by test and baseline primes in the different conditions of experiment 2**.

**Condition**	**Test**	**Baseline**	**Priming**	**Error in Test**	**Error in Baseline**
+WP, Full Prime	546 (46)	579 (74)	33	4.26	3.49
+WP, Partial Prime	595 (67)	598 (75)	03	3.29	3.49
+Phon, Full Prime	604 (84)	585 (81)	−19	4.26	2.71
+Phon, Partial Prime	593 (79)	602 (81)	9	4.26	2.33

The main effect of condition was significant [*F*_1(1, 85)_ = 10.89, *p* < 0.001; *F*_2(1, 47)_ = 3.52, *p* < 0.05] as was that of Prime Length [*F*_1(1, 85)_ = 5.81, *p* < 0.05; *F*_2(1, 47)_ = 4.31 The main effect of Prime Type was not significant [*F*_1_ and *F*_2_ < 1]. Condition interacted significantly with Prime Length [*F*_1(1, 85)_ = 17.55, *p* < 0.001; *F*_2(1, 47)_ = 6.95, *p* < 0.05] reflecting the fact that the partial prime and the full prime had opposite effects in the morphological and phonological conditions. The critical three-way interaction between Condition, Prime Length and Prime Type [*F*_1(2, 85)_ = 15.16, *p* < 0.001; *F*_2(2, 47)_ = 5.27, *p* < 0.05] indicated that priming was not constant across conditions. Further planned comparisons using Bonferroni protection levels at 0.05, showed that (a) priming effects were significant only in the [+WP, Full Prime] condition, [*F*_1(1, 85)_ = 6.74, *p* < 0.05; *F*_2(1, 23)_ = 5.55, *p* < 0.05] and the [+Phon, Partial Prime] condition [*F*_1(1, 85)_ = 5.24, *p* < 0.05; *F*_2(1, 23)_ = 5.35, *p* < 0.05], and (b) that these two conditions differed significantly from the [+WP, Partial Prime] and the [+Phon, Full Prime] conditions, but not form each other [*F*_1_ < 1, *F*_2_ < 1]. Similar analyses of the error data revealed no significant main effects or interactions.

Finally, to check on the possible contribution of the relevant stimulus properties (duration of the prime, and degree of phonemic overlap between prime and target), these two variable were centered and used in a separate stepwise regression analysis to determine the extent to which they modulate priming. Neither variable significantly predicted priming [prime duration: *R*^2^ = 0.00, *F*_(1, 94)_ = 0.36, *p* = 0.84; phonemic overlap: *R*^2^ = 0.02, *F*_(1, 94)_ = 2.54, *p* = 0.11].

These results are in keeping with the predictions developed within the CV-hypothesis. Priming by full word patterns is expected on this account since this is a structural unit that allows the drawing of generalization about the phonological structure and morpho-syntactic function of words. Also consistent with this view is the absence of facilitation by partial primes and by phonologically related primes both full and partial. In the case of partial primes, not enough information is provided about the phonological structure of the word, let alone its morpho-syntactic function, so no specific word pattern can be extracted and no savings can be made on the processing of the target. The full phonological prime is treated as a competitor since it is comprised of a root and a pattern that are different from those of the target (Frauenfelder et al., 2001).

## General discussion

The results of this study are consistent with the claims of the CV-hypothesis that consonants and vowels need to be segregated both in terms of representation and processing. More importantly, this study extends the CV-hypothesis in a significant way. In terms of processing dynamics this study shows that there is a differential time course at which information about consonants and vowels is mapped onto internal representations. Specifically, partial information about consonants (roots) is continuously used to generate lexical hypotheses, while partial information about vowels (word patterns) is ineffective in accessing the lexicon; only full information about the word pattern provides a basis for lexical access.

The differential processing mechanisms engaged by consonants and vowels suggest that access to information conveyed by consonants (roots) precedes access to information conveyed by vowels (patterns). This is consistent with what we know from masked priming and neuro-physiological research which clearly suggest that the lexical access process in Arabic is oriented toward the consonants (roots) (Boudelaa and Marslen-Wilson, 2005; Boudelaa et al., 2009). These differential processing dynamics arguably originate in the distinct function that consonants and vowels fulfill (McCarthy, 1981; Nespor and Vogel, 1986). Across languages, consonants convey constraining lexical information, and Semitic languages are a clear case where lexical meaning is the domain of the consonantal root. Since the task of the listener is to use the speech input to access meaning, a successful heuristic in Semitic languages is to rely on the consonants of the root. Vowels on the other hand primarily carry phonological and morpho-syntactic information. Apparently this information does not become available unless the full vowel pattern is heard.

It could be argued that the different processing time courses for consonants and vowels in Arabic stems simply from the fact that there are almost 5 times as many consonants in this language (28 consonants) as there are vowels (6 vowels). Based on this simple fact, individual consonants should narrow down the range of possible words more than vowels, and consequently the lexical access process would be oriented toward consonants (roots). However, this distributional bias in favor of the consonants should in fact result in their taking more time to recognize. If we assume, for the sake of argument, that the probability of a given consonant in the language is 1/28 and the probability of a given vowel is 1/6, the chances of making a correct guess regarding the identity of particular segment are much higher for vowels than consonants. Thus, the statistical distribution of the sounds of the language alone, leads us to expect vowels to be easier to recognize and more readily to project onto internal lexical representations. This is obviously not the case; consequently a statistical explanation of the present results is not viable.

In terms of the architecture of the lexicon, the results establish that vowels and consonants are represented independently, not at peripheral levels of modality specific representation as is the case with Indo-European languages (Caramazza et al., 2000; Carreiras et al., 2007, 2009), but more significantly at higher levels of the language processing system since this is the only site where the processing of an auditory prime can have any processing consequences for a visual target (Marslen-Wilson et al., 1994). Maintaining such distinct representations for consonants and vowels at higher levels of the system makes sense in the context of Semitic languages given the important functional implications such a distinction has for various domains of knowledge such as semantics and morphology and syntax.

The differences between vowels and consonants at the processing and representational levels in this study are at least in part due to the morphemic status that these elements play in the language. Consonants and vowels in Arabic are not simply distinct classes of phonemes, but morphemes with overt implications for semantic meaning and morpho-syntactic fucntions. However, a potential problem with this interpretation is that although roots are exclusively made up of consonants and word patterns are essentially made up of vowels, several Arabic word patterns feature a subset of consonants along with the vowels (e.g., {ma- -a-} *place noun*, or {muta-aa-i-} *agent noun*). So what processing mechanism applies to consonants that are part of the word pattern? How does the system determine that the consonant /m/ for instance is part of the word pattern in the word [masraħ] *theater*, but part of the root in the word [malak] *king*? Pilot data using words starting with a consonant that is either part of the pattern as in [masraħ] *theater* or part of the root as in [malak] *king* suggest that the language processor initially treats the sound /m/ similarly in the two words. It uses it in combination with other consonants in the input to access the lexicon. The words [masraħ] *theater* and [malak] *king* seem to activate the same set of candidates initially, but as more input is accumulated, different sets of candidates become more viable and get activated accordingly (Magnuson et al., 2007; Jesse and Massaro, 2010). This means that consonants are processed continuously and vowels discontinuously until the correct components of the word, that is the correct root and word pattern, are extracted.

For any model to accommodate the effects of continuous mapping of consonants and discontinuous mapping of vowels it must distinguish between these two elements in terms of representation and processing mechanisms as suggested by the CV-hypothesis, and elaborated here. Independently represented consonants (i.e., roots) act as direct targets for speech input in order to support the continuous mapping necessary for immediate access and efficient communication, while independently represented patterns of vowels will modulate the interpretation of the utterance at later processing stages.

## Implications for education and rehabilitation

The dissociation between consonants (roots) and vowels (word patterns) in the context of Arabic has important potential consequences for language practitioners in neuro-rehabilitation and neuro-education. Educators can make curricular changes based on this and similar studies by designing teaching materials where the distinction between the consonants (roots) and vowels (word patterns) and their functions is brought to the fore such that the language learner can build an awareness of these elements. This awareness can play an important role in helping not only unimpaired learners, but also children whose learning disabilities stem from deficiencies in metalinguistic skills (Bialystok et al., 2014; Tong et al., 2014). In this respect, a recent study by Kim et al. (2013) suggests that awareness of various linguistic domains such as phonology, orthography and morphology provide an effective predictor of reading abilities. More relevantly, morphological awareness generally defined as the child's conscious ability to reflect on and manipulate the structure of his/her language (Carlisle, 1995, p. 194) has been shown to be strongly associated with the child's reading development in languages such as English (Carlisle, 2000), French (Casalis and Louis-Alexandre, 2000) and Chinese (Ku and Anderson, 2003). The present study suggests that in the end-state mental lexicon of Arabic speakers consonants and vowels have different roles by virtue of the different morphemes with which they are typically associated. Language practitioners can capitalize on this finding and develop research informed syllabi that promote awareness of consonants and vowels as different morphemes. Consciously knowing how morphemes fit together and what kind of information they convey should facilitate the acquisition of reading as well the reading of novel words. A child who has a good grasp of the functional properties of consonants (roots) and vowels (word patterns) in Arabic will be better able to figure out the meaning of items like [haasuub] *computer* and [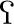
awlama] *globalize* when he/she first hears them. It will be relatively apparent for this child that the form [haasuub] breaks into the consonantal root {hsb} with the general meaning of *counting* and the vowel pattern {-aa-uu-} with a *singular noun* meaning because the child consciously knows what roots and patterns are and they have experienced the same morphological elements in other contexts such as [hisaab] *counting/calculus*, [mahsuub] *counted* etc…

In terms of the rehabilitory implications of the current study, the development of diagnostic test batteries and therapeutic methods should be guided by the present (and earlier) results. This paper offers an account of how Arabic consonants (roots) and vowels (word patterns) are differentially mapped onto internal representations of form and meaning. If such a model is accepted, then the language processing system can malfunction only in certain ways (Bullinaria and Chater, 1995; Pulvermüller et al., 2001; Small, 2004). One of these for instance is that in Arabic consonants and vowels can be selectively impaired and spared. Alternatively, deficits in the processing of the consonants of the root may co-occur with semantic deficits since the consonants convey meaning, whereas deficits in vowel processing should ally themselves with problems at the phonological level. This suggests that an effective aphasia test battery for Arabic needs not only to weight morphology as a domain of knowledge that is distinct from other domains, but it also needs to acknowledge the differential properties of different morphemes (i.e., root consonants and vowel patterns) in the processing and representation of the Arabic language. Failing this, the test may not be able to detect the patterns of selective deficits predicted by the model. In conclusion, the development of test batteries to assess acquired or developmental disorders of Arabic should do so in the context of emerging research findings, such as those reported here, about the specific properties of Arabic as a psycholinguistic system.

### Conflict of interest statement

The author declares that the research was conducted in the absence of any commercial or financial relationships that could be construed as a potential conflict of interest.

## Footnotes

^1^Although the root is exclusively consonantal, the word pattern may feature a subset of consonants along with its vowels.
